# Profiling of tRNA Halves and YRNA Fragments in Serum and Tissue From Oral Squamous Cell Carcinoma Patients Identify Key Role of 5′ tRNA-Val-CAC-2-1 Half

**DOI:** 10.3389/fonc.2019.00959

**Published:** 2019-09-26

**Authors:** Joseph Dhahbi, Yury O. Nunez Lopez, Augusto Schneider, Berta Victoria, Tatiana Saccon, Krish Bharat, Thaddeus McClatchey, Hani Atamna, Wojciech Scierski, Pawel Golusinski, Wojciech Golusinski, Michal M. Masternak

**Affiliations:** ^1^Department of Medical Education, School of Medicine, California University of Science & Medicine, San Bernardino, CA, United States; ^2^Translational Research Institute for Metabolism and Diabetes, AdventHealth, Orlando, FL, United States; ^3^Faculdade de Nutrição, Universidade Federal de Pelotas, Pelotas, Brazil; ^4^Burnett School of Biomedical Sciences, College of Medicine, University of Central Florida, Orlando, FL, United States; ^5^Department of Otorhinolaryngology and Laryngological Oncology in Zabrze, Medical University of Silesia, Katowice, Poland; ^6^Department of Otolaryngology and Maxillofacial Surgery, University of Zielona Gora, Zielona Gora, Poland; ^7^Department of Biology and Environmental Studies, Poznan University of Medical Sciences, Poznań, Poland; ^8^Department of Head and Neck Surgery, Poznan University of Medical Sciences, The Greater Poland Cancer Centre, Poznań, Poland

**Keywords:** 5′ tRNA halves, 5′ YRNA fragments, oral cancer, OSCC, small RNA-Seq, microRNA, coexpression network, WGCNA

## Abstract

Oral squamous cell carcinoma (OSCC) is the most common type of head and neck cancer and, as indicated by The Oral Cancer Foundation, kills at an alarming rate of roughly one person per hour. With this study, we aimed at better understanding disease mechanisms and identifying minimally invasive disease biomarkers by profiling novel small non-coding RNAs (specifically, tRNA halves and YRNA fragments) in both serum and tumor tissue from humans. Small RNA-Sequencing identified multiple 5′ tRNA halves and 5′ YRNA fragments that displayed significant differential expression levels in circulation and/or tumor tissue, as compared to control counterparts. In addition, by implementing a modification of weighted gene coexpression network analysis, we identified an upregulated genetic module comprised of 5′ tRNA halves and miRNAs (miRNAs were described in previous study using the same samples) with significant association with the cancer trait. By consequently implementing miRNA-overtargeting network analysis, the biological function of the module (and by “guilt by association,” the function of the 5′ tRNA-Val-CAC-2-1 half) was found to involve the transcriptional targeting of specific genes involved in the negative regulation of the G1/S transition of the mitotic cell cycle. These findings suggest that 5′ tRNA-Val-CAC-2-1 half (reduced in serum of OSCC patients and elevated in the tumor tissue) could potentially serve as an OSCC circulating biomarker and/or target for novel anticancer therapies. To our knowledge, this is the first time that the specific molecular function of a 5′-tRNA half is specifically pinpointed in OSCC.

## Introduction

Head and neck squamous cell carcinomas (HNSCC) are a type of malignant tumors located in the epithelium covering the upper aerodigestive tract (1). HNSCC most commonly occurs at the oral cavity (OSCC), is most prevalent in older patients (2), and is strongly associated with tobacco and alcohol consumption (3). HPV infections, on the other hand, account for up to 25% of all HNSCC cases and up to 60% of the oropharyngeal carcinoma subset (4–6). Accumulating evidence has linked the presence of specific non-coding RNAs to cancer diagnosis and prognosis (7–11) and underscored their role as potential regulators of tumorigenesis (12–14). MicroRNAs (miRNAs) are potent regulators of gene expression and are currently the most studied sncRNAs (15). Circulating miRNAs found in a variety of bodily fluids have been demonstrated to have excellent potential as biomarkers of diseases including cancer (11, 16–18) and of physiological conditions such as aging, calorie restriction and dwarfism (19–22). Tumor-derived miRNAs can regulate the expression of oncogenes and tumor suppressor genes in the local microenvironment (7, 23) and by traveling in the circulation as complexes with other proteins and lipids or inside exosomes, can also contribute to priming distant tissues for metastasis (24–27).

Small RNA sequencing used in our previous and recent work revealed significant association between pathologic and physiologic states and changes in the expression of sncRNAs derived from tRNAs and YRNAs (17, 19, 21, 28–33). Additional evidence supports an active role for these sncRNAs in cell function, disease development and progression (34–38). Importantly, we have previously reported that serum levels of 5′ tRNA halves and 5′ YRNA fragments are altered in patients with breast cancer and head and neck squamous cell carcinoma (17, 31). More recently, we found that levels of 5′ tRNA halves change in seminal fluid and tumor tissues from patients with prostate cancer (33). We also previously presented evidence that aging, calorie restriction and dwarfism altered the expression of circulating 5′ tRNA halves in addition to miRNAs (20, 29, 30). In the present study, we report that serum and tumor tissue levels of 5′ tRNA halves and 5′ YRNA fragments change in association with an OSCC diagnosis. Additionally, we suggest novel cancer-related functions for the 5′ tRNA-Val-CAC-2-1 half.

## Results

### tRNA Halves and YRNA Fragments Detected in the Human Circulation and Tissue Are Mostly Derived From the 5′-End of the Respective Full-Length Molecule

To investigate sncRNAs as potential circulating biomarkers of OSCC, we used small RNA-Seq to measure differential expression of tRNA- and YRNA-derived small RNAs in serum samples collected from subjects without known cancers and patients with OSCC. To reveal whether tRNA- and YRNA-derived small RNAs are also differentially expressed in solid OSCC tissue besides serum, we analyzed a small RNA-Seq generated from tumor and matched normal tissues. We have previously found in our studies, that circulating tRNA- and YRNA-derived small RNAs are 30–34 nucleotides long and almost all of the sequencing reads map to the 5′ end of tRNA or YRNA genes (17, 28, 29, 31, 33). Once again, we find in the present study that 99.97% of the reads with length 30–34 nt derived for tRNA align with the 5′ end of tRNA genes. Similarly, 99.93% of the reads with length 30–34 nt derived for YRNAs align with the 5′ end of YRNA genes. Hence, only reads that are 30–34 nucleotides long and mapping to the 5′ end of tRNA or YRNA genes were used for differential expression analysis in this study.

### Levels of 5′ tRNA Halves Decreased in Serum of HNSCC Cancer Patients

**Figure 1:**
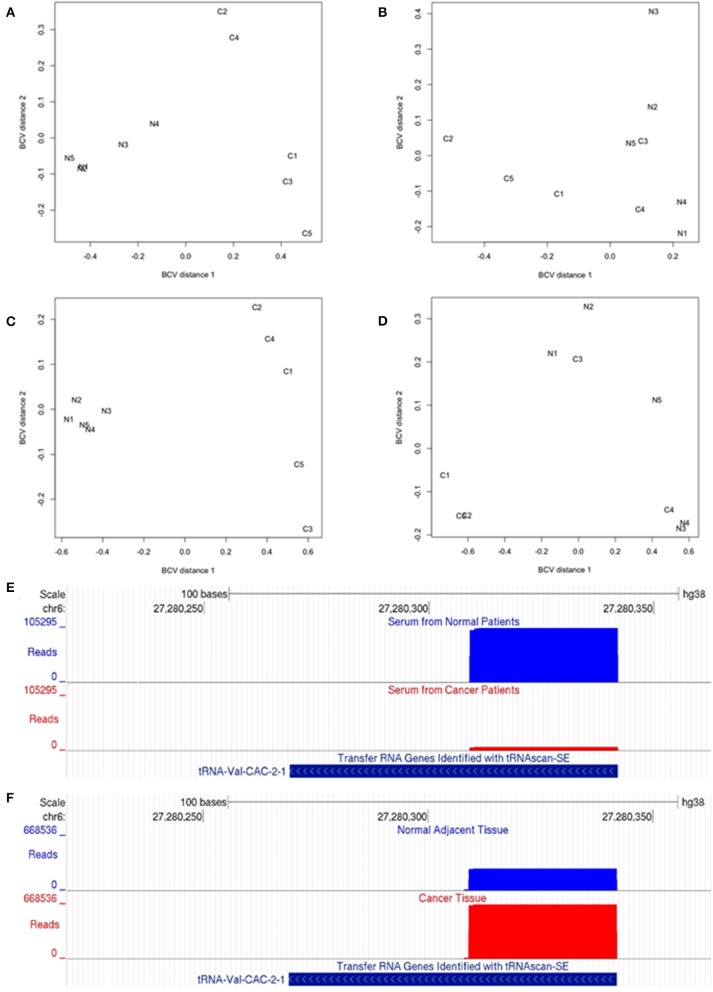
Small non-coding RNA-Seq analysis in OSCC. **(A–D)** Multi-dimensional scaling (MDS) analysis of the expression levels of 5′ tRNA-halves and 5′ YRNA fragments. The MDS analysis generates distances that represent the biological coefficient of variation (BCV) between samples. MDS plots show serum expression differences of 5′ tRNA-halves between patients with and without OSCC **(A)** and between tumor and adjacent normal tissues **(B)**. Also, MDS plots show serum expression differences of 5′ YRNA fragments between patients with and without OSCC **(C)** and between tumor and adjacent normal tissues **(D)**. Dimension 1 of the MDS plot depicts the cancer effect on the expression levels of 5′ tRNA-halves or 5′ YRNA fragments, while dimension 2 represents the homogeneity between biological replicates. Samples N1–N5 represent Normal while samples C1–C5 represent Cancer. **(E,F)** Differential expression of a 5′ tRNA-half derived from the tRNA-Val-CAC-2-1 gene in serum from patients with and without OSCC **(E)**, and in solid OSCC tumor relatively to adjacent normal tissue **(F)**. The UCSC genome browser screenshots illustrate the alignment of reads to the tRNA-Val-CAC-2-1 gene. **(E)** The alignment (number of reads, y-axis) shows that the numbers of reads mapping to the 5′ end of tRNA-Val-CAC-2-1 gene are significantly lower in serum from patients with (red) than without (blue) OSCC. **(F)** In contrast, the alignment shows that the numbers of reads mapping to the 5′ end of tRNA-Val-CAC-2-1 gene are significantly higher in solid OSCC (red) relatively to adjacent normal tissue (blue). Shown at the bottom is the tRNA-Val-CAC-2-1 gene annotation from the tRNA genes track “Transfer RNA Genes Identified with tRNAscan-SE” associated with the human GRCh38/hg38 genome.

We compared the levels of 5′ tRNA halves in serum from subjects with and without OSCC using the edgeR exact test which is applicable to experiments with a single factor (39). We first assessed the biological coefficient of variation (BCV) between samples and found that the cancer samples are markedly separated from the normal samples (Figure 1A, BCV distance 1). Moreover, the normal samples appear more homogeneous than the cancer samples (Figure 1A, BCV distance 2). The differential expression analysis revealed that OSCC decreased the serum levels of 22 types of 5′ tRNA halves with average read counts-per-million (CPM) > 1,770, fold decrease > 5, and FDR < 0.01% (Table 1). The decreased 5′ tRNA halves are derived from five isodecoders of tRNA-Arg, -Glu, -His, -Lys, and -Val. We then examined the tissue expression levels of these 22 types of 5′ tRNA halves and found that they are substantially abundant in tumor and normal tissues (Table 1; see CPM under “Tumor tissues”). However, only one of the 22 types of 5′ tRNA halves (i.e., the 5′ tRNA half derived from the tRNA-Val-CAC-2-1 gene) significantly (FDR < 5%) changed expression in both serum and tissue (Table 1, Figures 1E,F).

**Table 1:** Twenty-two 5′ tRNA halves significantly (FDR < 5%) decreased in serum of patients with OSCC.

**^a^tRNA gene name**	**^b^Genomic coordinates and strand**	**Serum**				**Tumor tissues**			
		**^c^CPM**	**^d^FC**	**^d^*P*-value**	**^d^FDR (%)**	**^c^CPM**	**^d^FC**	**^d^*P*-value**	**^d^FDR (%)**
tRNA-Arg-TCT-1-1	chr1:93847572-93847657 +	3191	−6.1	<0.001	<0.001	73	3.6	0.004	12
tRNA-Glu-TTC-2-1	chr13:44917926-44917998 –	1774	−6.6	<0.001	<0.001	600	1.4	0.388	77
tRNA-Glu-TTC-2-2	chr15:26082233-26082305 –	1780	−6.8	<0.001	<0.001	591	1.4	0.373	77
tRNA-His-GTG-1-5	chr6:27158126-27158198 +	1810	−6.7	<0.001	<0.001	1,374	1.7	0.124	65
tRNA-Lys-CTT-1-1	chr14:58239894-58239967 –	2449	−5.1	<0.001	<0.001	3,226	−1.0	0.968	97
tRNA-Val-AAC-1-1	chr3:169772229-169772302 +	33416	−5.7	<0.001	0.001	54,730	1.4	0.013	23
tRNA-Val-AAC-1-2	chr5:181164153-181164226 +	34680	−5.7	<0.001	0.001	56,639	1.4	0.013	23
tRNA-Val-AAC-1-3	chr5:181169609-181169682 +	34102	−5.6	<0.001	0.001	55,723	1.4	0.013	23
tRNA-Val-AAC-1-4	chr5:181218269-181218342 -	9117	−5.8	<0.001	<0.001	14,817	1.5	0.064	53
tRNA-Val-AAC-1-5	chr6:27753399-27753472 –	8871	−5.7	<0.001	<0.001	14,333	1.4	0.074	53
tRNA-Val-AAC-3-1	chr6:27650927-27651000 –	8897	−5.9	<0.001	<0.001	14,112	1.4	0.074	53
tRNA-Val-AAC-4-1	chr6:27681105-27681178 –	8658	−6.0	<0.001	<0.001	14,021	1.4	0.073	53
tRNA-Val-CAC-1-1	chr1:161399699-161399772 –	9451	−5.5	<0.001	0.001	15,785	1.4	0.107	61
tRNA-Val-CAC-1-2	chr5:181097069-181097142 +	34761	−5.4	<0.001	0.001	59,247	1.4	0.029	33
tRNA-Val-CAC-1-3	chr5:181102252-181102325 –	9028	−5.5	<0.001	0.001	15,380	1.4	0.105	61
tRNA-Val-CAC-1-4	chr5:181173649-181173722 +	34900	−5.5	<0.001	0.001	59,839	1.4	0.028	33
tRNA-Val-CAC-1-5	chr5:181222394-181222467 –	9298	−5.4	<0.001	0.001	15,538	1.4	0.097	59
tRNA-Val-CAC-1-6	chr6:26538053-26538126 +	34603	−5.5	<0.001	0.001	58,857	1.4	0.029	33
tRNA-Val-CAC-4-1	chr1:143803993-143804066 –	9212	−5.6	<0.001	<0.001	15,155	1.4	0.106	61
tRNA-Val-CAC-5-1	chr1:121020728-121020801 –	8863	−5.6	<0.001	0.001	14,921	1.4	0.107	61
tRNA-Val-CAC-chr1-93	chr1:149712551-149712624 –	9465	−5.5	<0.001	0.001	15,726	1.4	0.098	59
^e^tRNA-Val-CAC-2-1	chr6:27280269-27280342 –	17293	−7.6	<0.001	<0.001	39,336	2.0	<0.001	1

a *tRNA gene name from Genomic tRNA Database (gtrnadb.ucsc.edu)*.

b *Genomic coordinates of tRNA genes in the human GRCh38/hg38 genome*.

c *Average tRNA read counts-per-million (CPM) computed over all libraries from serum or tissue taking into account the estimated dispersions and the libraries sizes. It represents a measure of the overall expression level of the tRNA fragments*.

d *Fold change, P-value and FDR (<5%) for differential abundance computed by EdgeR*.

e *tRNA half that changed expression in both tumor tissues and serum*.

*Respective tumor tissue data presented for comparison*.

### Levels of 5′ tRNA Halves Increased in OSCC Tissues Relatively to Adjacent Healthy Tissues

Since most of the 5′ tRNA halves (21 out of 22) that decreased expression in the serum of cancer patients did not significantly change in solid tumor tissue, we sought to identify other types of 5′ tRNA halves that may change in solid tumor tissue independently of the changes in the serum levels. We used a statistical test appropriate for paired designs in EdgeR (39) to analyze the expression of 5′ tRNA halves in tumor and matched normal tissues. Here, even though the biological coefficient of variation (BCV) showed separation between normal and cancer samples, the homogeneity between replicates was less marked than in the serum samples (Figures 1A,B). The differential expression analysis detected six 5′ tRNA halves that are highly expressed (CPM > 12,630) and significantly increased (Fold change ≥ 1.8 and FDR < 4.0%) in tumor compared to matched normal tissues (Table 2). These differentially expressed 5′ tRNA halves are derived from two isodecoders of tRNA-Gly and tRNA-Val. Upon examination of the serum levels of these six 5′ tRNA halves that increased expression in OSCC tissues, we found that all five 5′ tRNA halves derived from tRNA-Gly remain unchanged in the serum of cancer patients (Table 1). As reported above, only the 5′ tRNA half derived from tRNA-Val-CAC-2-1 gene significantly changed expression in both serum and solid tumor (Tables 1, 2). However, the expression changes of 5′ tRNA-Val-CAC-2-1 half are in opposite directions: it is upregulated in solid tumor and downregulated in serum (Figures 1E,F).

**Table 2:** Six 5′ tRNA halves significantly (FDR < 5%) increased in tumor relatively to healthy adjacent tissue in patients with OSCC.

**^a^tRNA gene name**	**^b^Genomic coordinates and strand**	**Tumor tissues**				**Serum**			
		**^c^CPM**	**^d^FC**	**^d^*P-*value**	**^d^FDR (%)**	**^c^CPM**	**^d^FC**	**^d^*P*-value**	**^d^FDR (%)**
tRNA-Gly-CCC-1-2	chr1:16861920-16861991 +	39,401	2.1	<0.001	0.716	118,338	1.2	0.584	68
tRNA-Gly-CCC-chr1-137	chr1:16545938-16546009 –	12,631	2.1	0.001	3.875	37,450	1.1	0.705	79
tRNA-Gly-GCC-2-5	chr16:70789506-70789577 +	46,189	1.8	<0.001	1.989	120,217	1.2	0.586	68
tRNA-Gly-GCC-2-6	chr17:8125745-8125816 +	46,245	1.8	<0.001	1.989	117,350	1.2	0.594	69
tRNA-Gly-GCC-5-1	chr16:70788693-70788764 +	45,777	1.8	<0.001	1.989	119,488	1.2	0.582	68
^e^tRNA-Val-CAC-2-1	chr6:27280269-27280342 –	39,336	2.0	<0.001	0.777	17,293	−7.6	<0.001	0

a *tRNA gene name from Genomic tRNA Database (gtrnadb.ucsc.edu)*.

b *Genomic coordinates of tRNA genes in the human GRCh38/hg38 genome*.

c *Average tRNA read counts-per-million (CPM) computed over all libraries from serum or tissue taking into account the estimated dispersions and the libraries sizes. It represents a measure of the overall expression level of the tRNA fragments*.

d *Fold change, P-value and FDR (<5%) for differential abundance computed by EdgeR*.

e *tRNA half that changed expression in both tumor tissues and serum*.

*Respective serum data presented for comparison*.

### Levels of 5′ YRNA Fragments Decreased in Serum of OSCC Patients But Did Not Significantly Change in Solid Tumor Relatively to Adjacent Healthy Tissue

Similar to the analysis for 5′ tRNA halves, we compared the levels of 5′ YRNA fragments in serum from subjects with and without cancer and found marked separation between normal and cancer samples (Figure 1C, BCV distance 1) and adequate homogeneity of the replicates (Figure 1C, BCV distance 2). The differential expression analysis revealed that OSCC decreased the serum levels of 4 types of 5′ YRNA fragments with average read CPM > 150,000, a fold decrease > 2.5, and FDR < 0.01% (Table 3). In contrast, we found no significant separation between normal and cancer samples (Figure 1D) and no significant differential expression for these specific four types of 5′ YRNA fragments between tumor and matched normal tissues.

**Table 3:** 5′ YRNA fragments significantly changed in serum of patients with OSCC.

**^a^YRNA gene name**	**^b^Genomic coordinates and strand**	**Serum**			
		**^c^CPM**	**^d^FC**	**^d^*P*-value**	**^d^FDR**
RNY4-201	chr7:148963314-148963410 +	333105	−2.7	<0.001	0.002%
Y_RNA.295-201	chr3:157153547-157153640 +	331916	−2.7	<0.001	0.003%
RNY4P10-201	chr6:33199600-33199696 +	327747	−2.6	<0.001	0.003%
RNY4P7-201	chr2:127798902-127798998 –	151705	−2.8	<0.001	0.001%

a *YRNA gene name from the UCSC GENCODE v24 track*.

b *Genomic coordinates of the YRNA genes in the human GRCh38/hg38 genome*.

c *Average tRNA read counts-per-million computed over all libraries and taking into account the estimated dispersions and the libraries sizes. It represents a measure of the overall expression level of the tRNA fragments*.

d *Fold change, P-value and FDR (<5%) for differential abundance computed by EdgeR*.

### 5′ tRNA-Val-CAC-2-1 Half Is Part of a Key miRNA-Enriched Regulatory Coexpression Module That Highly Significantly Correlate With the Cancer Trait

To gain additional insight into potential functions of the 5′ tRNA halves and 5′ YRNA fragments, we implemented a novel approach based on weighted gene coexpression network analysis (WGCNA) of the pooled small RNA-Seq data including 5′ tRNA halves (this study), 5′ YRNA fragments (this study) and miRNAs from the same samples as previously published (18). WGCNA was originally developed to build gene networks based on a gene coexpression measure that describes the correlation among genes with similar patterns of expression (40, 41). Those sets of genes with similar (correlated) expression patterns are denominated modules and represent non-linear pathways with specific biological function. Since its inception in 2005, this methodology has been applied mainly in the gene expression context, however, the applicability of the method has also been extended to the study of other genetic features such as protein, miRNA, and lncRNA networks (42–46). In this study, we further extend the applicability of WGCNA by applying it to sncRNA sequence data that includes 5′ tRNA halves, 5′ YRNA fragments, and miRNAs. We reasoned that miRNAs and 5′ tRNA halves and/or 5′ YRNAs fragments that are coregulated and involved in common biological pathways will belong to common coexpression modules detected by the WGCNA method. This would allow us, using a concept reminiscent of “guilt by association,” to assign/predict specific biological functions for novel cancer-relevant 5′ tRNA halves and 5′ YRNA fragments based on the larger body of knowledge accumulated for miRNAs.

**Figure 2:**
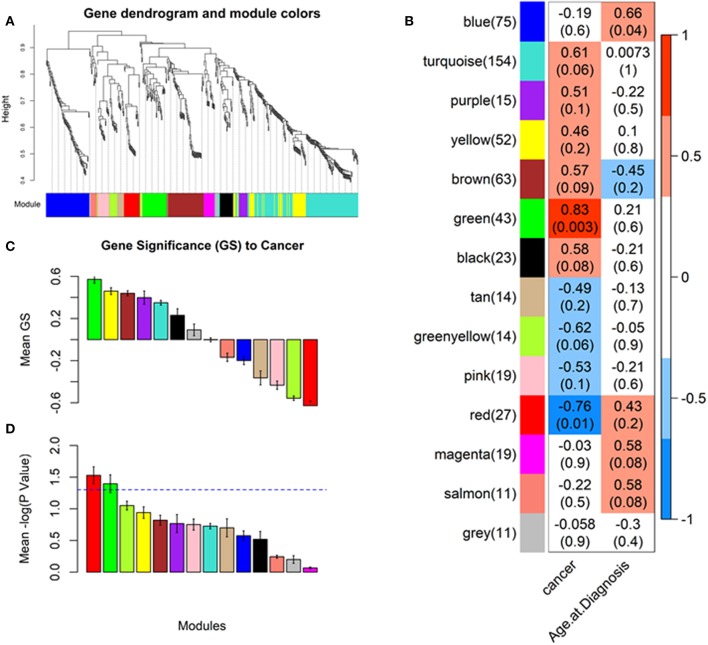
Identifying cancer-relevant modules by weighted gene coexpression network analysis (WGCNA). The network was created from the weighted correlation matrix generated by the WGCNA package in the R environment. First, an adjacency matrix is calculated, then the topological overlap (TO) to hierarchically cluster genes into coexpression modules (see section Materials and Methods). Final module assignments were made based on module membership. **(A)** Cluster dendrogram groups genetic features into distinct modules. The y-axis represents a dissimilarity distance (1–TO). Dynamic tree cutting was used to determine modules, by dividing the dendrogram at significant branch points (identifying cancer-relevant modules). **(B)** Correlations between the module eigengenes (representative module expression pattern) and the cancer tratit. Green and Red modules display strong, highly significant correlations to the cancer trait (*P*-values between parenthesis, scale bar represents the range of correlation coefficients). **(C)** Barplot of the average gene significance (GS) for each detected module, equivalent to the average correlation between the module genetic features and the cancer trait. **(D)** Barplot of the average –log *P*-value of GS. Two modules: Green and Red have a mean GS *P* < 0.05 (–log_10_(*P*) > 1.3). Color-coding of the plots' bars represent module names, unless specified as for the scale bar in **(B)**.

**Figure 3:**
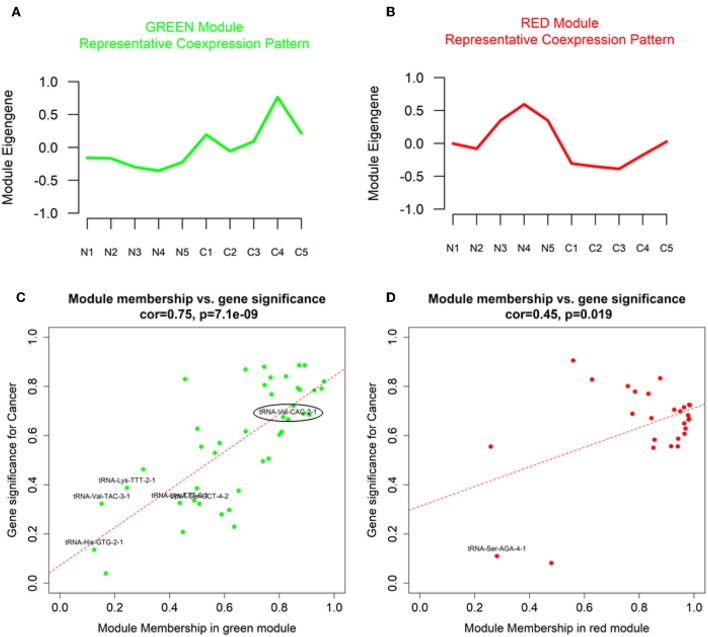
Characteristic coexpression patterns (module eigengenes), gene significance (GS) and module membership for genetic features in OSCC-relevant modules. **(A,B)** Plots of the module eigengenes (representative coexpression pattern) for the cancer-relevant Green and Red modules **(C,D)**. Scatter plots of correlations between gene significance (GS) and module membership for cancer-relevant Green and Red modules. Color-coding is equivalent to module names. Non-miRNA genetic features are labeled with black fonts in the scatter plots. Oval: most relevant 5′ tRNA half.

Our analysis identified a total of 14 coexpressed modules (Figures 2A–D). Two modules, namely Green (with 43 features upregulated in OSCC tissue) and Red (with 27 features downregulated in OSCC tissue), demonstrated a representative coexpressed pattern that significantly correlated with the cancer trait (positively for the Green module with r = 0.83, *P* = 0.003 and negatively for the Red, with *r* = −0.78, *P* = 0.01, Figures 2B, 3A,B). No association was detected for either the Green or the Red module with the age at diagnosis (Figure 2B). In addition, module membership (MM) of the individual genetic features in the Green module demonstrated a highly significant correlation with their respective gene significances (GS) for the cancer trait (*r* = 0.75, *P* = 7.1 × 10^−9^, Figure 3C). MM quantifies how coexpressed, in absolute terms, a given genetic feature (i.e., miRNA, 5′ tRNA halves, or 5′ YRNA fragments) is with respect to the other features in a particular module (47). The Green module mainly consisted of miRNAs but importantly included six 5′ tRNA halves, one of which (5′ tRNA-Val-CAC-2-1 half, shown enclosed in an oval in Figure 3C) displayed both high membership in the module (MM = 0.83) and high GS (GS = 0.67) to the cancer trait. Although to a lower extent, membership of the genetic features in the Red module also significantly correlated with the GS to the cancer trait (*r* = 0.45, *P* = 0.019, Figure 3D). The Red module was mainly composed of miRNAs (unlabeled dots in the plots), with a single 5′ tRNA half (labeled red dot) belonging to the module but with low module membership and low GS to the cancer trait (Figure 3D). No 5′ YRNA fragments were detected in any of the two modules with statistically significant correlations to the cancer trait. However, multiple other 5′ tRNA halves and 5′ YRNA fragments belonged to modules (i.e., Black, Brown, GreenYellow, and Turquoise) that displayed marginally significant trends (0.05 < *P* < 0.1) with the cancer trait (Figure 2B, Supplementary Figures 1, 2).

### 5′ tRNA-Val-CAC-2-1 Half Is Part of a Key miRNA-Enriched Regulatory Coexpression Module That Targets Genes Involved in the Negative Regulation of the G1/S Transition of the Mitotic Cell Cycle

As commented above, we reasoned that miRNAs and 5′ tRNA halves and/or 5′ YRNA fragments that are coregulated and involved in common biological pathways will belong to common coexpression modules detected by the WGCNA method. Significant coexpression among miRNAs and 5′ tRNA halves was evident in several modules detected by our analysis (modules containing multiple types of sncRNA, Figure 3, Supplementary Figures 1, 2). Because of its particular relevance to the cancer trait, we focused on the Green module to further explore the biological functions of the module and the “guilt by association” functions of the most relevant 5′ tRNA half belonging to this module (namely, the 5′ tRNA-Val-CAC-2-1 half). For this, we conducted “miRNA-overtargeting” analysis for the top 10 miRNAs (Supplementary Table 1) belonging to the Green module, which demonstrated high module membership and high gene significance to the cancer trait. By definition, significantly overtargeted genes in a particular network are genes that experimentally demonstrate significantly higher number of interactions with select miRNAs than expected by chance (48). By identifying enriched biological processes and functions for the network of significantly overtargeted genes, we could then assign specific functions to the related module(s) and to novel genetic features (e.g., 5′ tRNA halves and/or 5′ YRNA fragments) with high module membership. This analytical approach is supported by our previous experiences and those from others indicating that global miRNA-driven regulatory events commonly occur in a coordinated/cooperative fashion (17, 20, 42, 43, 48–52).

**Figure 4:**
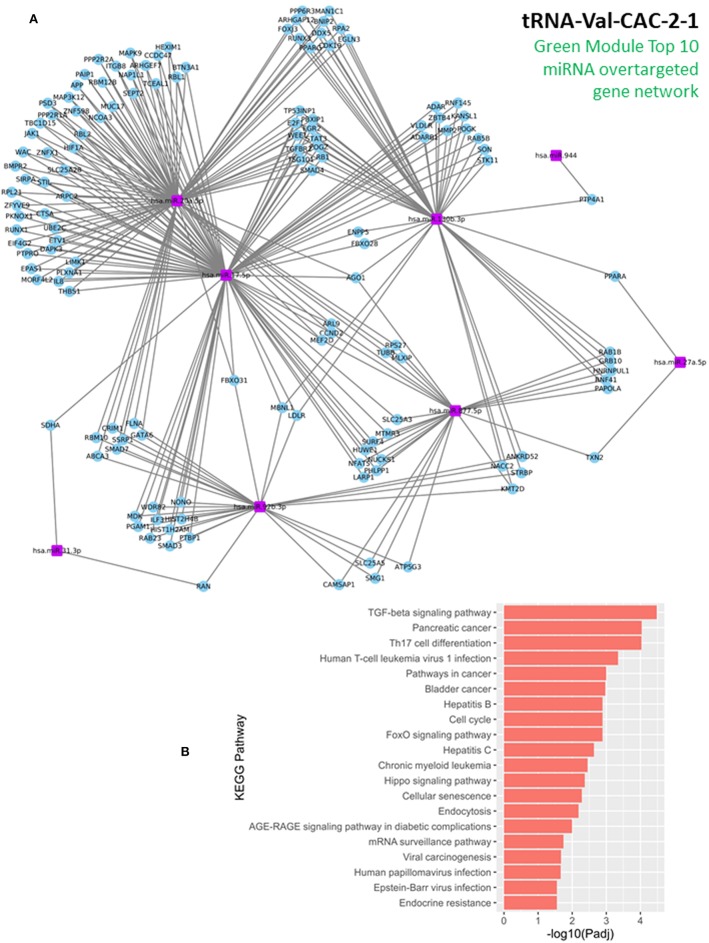
Discovering molecular functions for the cancer-associated Green coexpression module. **(A)** Network of genes that are significantly overtargeted by the top 10 miRNAs in the Green module. Blue circles represent overtargeted genes, which are potentially downregulated in tumor tissue (validation of gene expression subset in next figure). Purple rectangles represent the top 10 miRNAs with highest membership in the Green module and high gene significance to the cancer trait (these miRNAs are upregulated in the OSSC tissue). **(B)** KEGG pathway enrichment among the Green module-overtargeted genes shown in **(A)**. Companion table for the enrichment analysis is presented in Supplementary Table 3.

**Figure 5:**
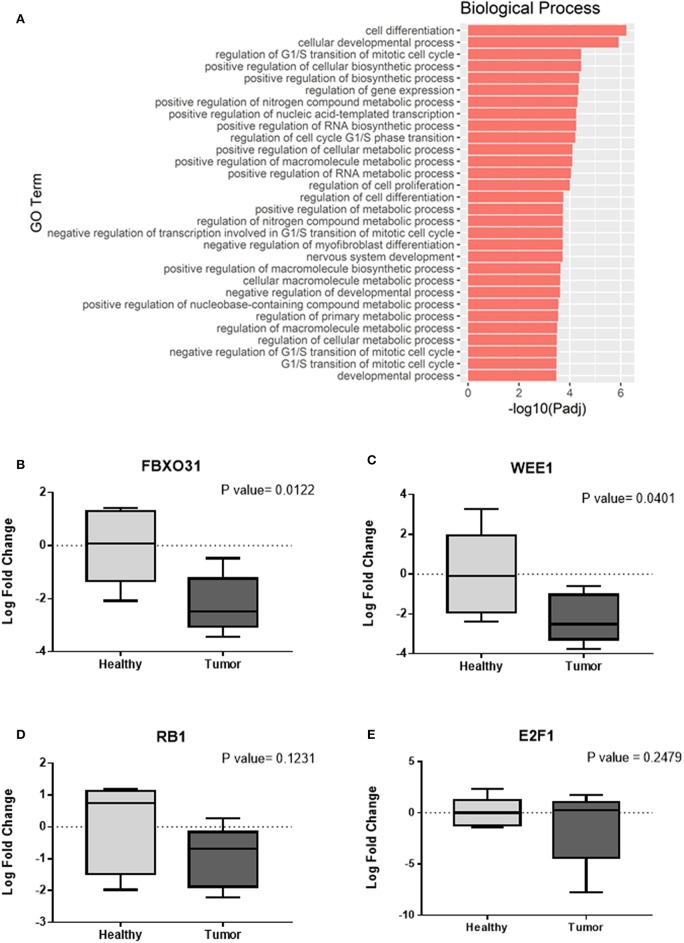
Confirming cell cycle-related functions for the cancer-associated Green coexpression module. **(A)** Enrichment for gene ontology (GO, biological processes) annotations among the Green module-overtargeted genes shown in Figure 4A. Companion table for the enrichment analysis is presented in Supplementary Table 4. **(B–E)** Validation of gene expression changes in tumor tissue (compared to adjacent normal/healthy tissue) by quantitative real time PCR (qPCR). Relative quantification was implemented using the 2^−ΔΔCt^ method for calculation of fold changes in gene expression. Expression of housekeeping gene β2-microglobulin was used as endogenous reference for normalization. Statistical analysis using the Student's *t*-test was implemented on the log transformed fold change data.

In this study, our analysis detected 135 validated miRNA-targeted genes that are significantly overtargeted by the set of top 10 miRNAs from the Green module (Figure 4A, Supplementary Table 2). Simulation analysis with 100,000 iterations demonstrated that finding such a number (or larger) of transcripts being significantly overtargeted by a set of 10 random miRNAs has a significantly low probability equal to 0.01137. This underscores the relevance of the specific gene network targeted by the cancer-associated Green module miRNAs and tRNA halves. KEGG pathway enrichment analysis on the list of targeted genes identified multiple cancer-related pathways, the cell cycle, and viral infection-related pathways among the most significantly enriched (P < 0.01, FDR < 0.05, Figure 4B, Supplementary Table 3). Interestingly, the enrichment in six viral-related pathways suggests a role for Green module miRNAs and tRNA-halves in the transduction of viral signals that may contribute to the development of OSCC. Supporting our reasoning, accumulating evidence confirm the virus-miRNA-cancer axis (53–59) and recent reports highlight the modulation of tRNA-halves in response to viral infections, including in the context of cancer (60–62). Similar to the KEGG pathway analysis, further analysis of gene ontology (GO) annotations for biological processes enriched among the significantly overtargeted genes highlighted the involvement of the Green coexpression module in the regulation of the cell cycle, specifically in the negative regulation of the G1/S transition of the mitotic cell cycle, and in negative regulation of cell differentiation (Figure 5A, Supplementary Table 4). Key cell cycle and differentiation-related transcripts regulated by members of the Green module are: WEE1, E2F1, RB1, and FBXO31 (Figure 4A, Supplementary Table 4). Remarkably, we were able to experimentally validate the results from our bioinformatic analysis. Using qPCR on tissue RNA, we demonstrated the significant downregulation of FXBO31 and WEE1 (Figures 5B,C) in the tumor tissue compared to healthy adjacent mucosal tissue. Although non-significantly, RB1 and E2F1 also displayed downregulation trends (Figures 5D,E). An additional interesting observation from our network analysis is that the AGO1 transcript is significantly overtargeted by the larger number of cancer-correlated miRNAs (AGO1 is targeted by four out of the top 10 Green module miRNAs, Figure 4A, Supplementary Table 2).

## Discussion

The association between changes in the expression of 5′ tRNA halves and 5′ YRNA fragments and cancer are currently largely descriptive. It is still unclear how changes in the expression of these unusual sncRNAs contribute to the process of carcinogenesis. Nonetheless, there are instances where the abundance and function of particular tRNA halves have been linked to cancer (17, 31, 33, 34, 37, 38). In this study, we are able to observe more changes in 5′ tRNA halves in serum than in tissue samples, indicating a wide array of circulating 5′ tRNA halves available for biomarker investigations in cancer patients.

Interestingly, we found that 5′ tRNA-Val-CAC-2-1 half significantly changed expression in both serum and tissue samples, although it was down-regulated in serum and up-regulated in tissue samples. Similar to 5′ tRNA halves, the 5′ YRNA fragments were more affected in serum than solid tissue samples, further confirming their suitability for blood-based diagnosis. We also found that the 5′ tRNA-Val-CAC-2-1 half belonged to a key miRNA-enriched regulatory coexpression module (denominated Green module) that was upregulated in the cancer tissue and targets genes involved in the negative regulation of the G1/S transition of the mitotic cell cycle and cell differentiation, namely FBXO31, WEE1, RB1, and E2F1. We also noticed in the network of Green module-overtargeted genes that AGO1 was the transcript overtargeted by the larger number of cancer-correlated miRNAs, which suggest a central, hub-like role for this gene in mediating the network effects of the Green module. Interestingly, AGO1 has been reported to directly bind tRNA precursors and to be potentially involved in the biogenesis of a subset of 5′ tRNA-derived fragments. Such 5′ tRNA fragments were predicted to be involved in gene silencing (63). More recently, tRNA fragments that act as tumor suppressors were reported to downregulate RNA-binding protein YBX1, which consequently led to destabilization of many pro-oncogenic transcripts (64). Our results now suggest pro-oncogenic 5′ tRNA half-mediated regulation in the opposite direction. Altogether, the evidence suggests that the miRNAs and 5′ tRNA halves (in particular 5′ tRNA-Val-CAC-2-1 half) belonging to the oncogenic Green module may function, at least in part, by downregulating the expression of AGO1 and consequently downregulating the expression of a subset of tumor suppressors 5′ tRNA halves. Therefore, our results and the existent literature support the notion that cancer regulatory mechanisms involve the mutually exclusive action of oncogenic and tumor suppressor subsets of sncRNAs comprised of coregulated miRNAs and 5′ tRNA halves, among other genetic players. Notably, the transcriptional regulatory activity of the oncogenic Green module, including 5′ tRNA-Val-CAC-2-1 half, appears to result in the activation of the cell cycle by downregulating the expression of genes involved in the negative regulation of the G1/S transition (downregulation of these genes in our study samples was experimentally confirmed by qPCR). Supporting our findings, Green module-overtargeted FBXO3 has been indeed reported to be downregulated in multiple cancers and to suppress cell proliferation partly through the degradation of cyclin D1 (65). On the other hand, the nuclear protein Wee1 (the product of the Green module-overtargeted WEE1 gene) was recently recognized as a tumor suppressor that regulates the progression of pancreatic cancer cells via delaying G2, which allows for longer time to repair post-replication errors in genomically unstable cancers (66). Similarly, the RB1 gene is mutated in a variety of human cancers and represents a classical prototype of tumor suppressor, inhibiting cell proliferation primarily through targeting of the E2F transcription factor and, additionally, through transcription-independent mechanisms (67). Although the E2F is hyperactive in many human cancers, its effects are reported to be context dependent, dichotomous and contradictory in almost all cases (68). Supporting our results, Zhong and colleagues recently reported that loss of E2F1 improves survival and accelerates oral tumor growth in mice (69). The tumor-suppressive effect of E2F1 has been ascribed to occur at the cancer promotion stage and may involve the induction of autophagy and apoptosis (68, 70).

Additional evidence support the role of tRNA-derived fragments, including 5′ tRNA halves, in the regulation of mRNA stability and translation, thereby affecting cancer cell proliferation and metastasis (64, 71–73). For example, 5′ tRNA halves induced by hypoxic stress were shown to suppress breast cancer metastasis, which was further shown to only proceed when specific 5′ tRNA halves were antagonized (73). Likewise, a specific 5′-half of tRNA-Asp-GUC was shown to be required for the proliferation of prostate carcinoma cells and knockdown of the 5′ tRNA-Asp-GUC half, but not its 3′ counterpart, interfered with cell proliferation (74). Moreover, dysregulation of other 5′ tRNA halves has been correlated with cancer staging and grading levels (73, 75). Expression of 5′ tRNA-Leu-CAG half was altered in tumor tissue and serum from patients with non-small cell lung cancer with a positive correlation between tumor development stage and the 5′ tRNA half expression levels (73). Expression of 5′ tRNA-4-Val-AAC was altered in renal cell carcinoma tissue samples with inversed correlation between expression levels and tumor staging and grading (75). It was also reported that specific 5′ tRNA halves were produced more abundantly in androgen receptor (AR)-positive prostate cancer and in estrogen receptor (ER)-positive breast cancer than in the respective negative forms (74). That investigation demonstrated that biogenesis of specific 5′ tRNA halves may be sex hormone-dependent (74). Additionally, given the well-established role of sex hormones in the control of proliferation and progression of prostate and breast cancers (76, 77), the observed link between the biogenesis of 5′ tRNA halves and alterations in the abundance of sex hormones may imply that 5′ tRNA halves take part in tumorigenesis of hormone-sensitive cancers. Similarly, we previously reported a significant association between alterations in the circulating levels of specific 5′ tRNA halves and the ER status and other clinicopathologic characteristics of breast cancer including PR, HER2, inflammation, and subsequent relapse (31).

In addition to our findings in this study that serum levels of 5′ YRNA fragments change in patients with OSCC, we have previously reported significant changes in serum and tissues levels of specific YRNA-derived fragments in patients with breast cancer (31) and in serum of patients with HNSCC (17). This association between changes in the abundance of YRNA-derived fragments and cancer suggests a possible function related to carcinogenesis. Supporting evidence includes not only the presence of YRNAs or their derivatives in several types of cancer (78, 79), but also their involvement in processes closely related to cancer such as cell proliferation (80, 81), apoptosis (82–84), senescence (85–87), and stress responses (88). Moreover, it was suggested that YRNA fragments may play a role in the mechanisms by which cancer cells establish a favorable microenvironment for proliferation and invasion (83). Furthermore, the expression of full length YRNAs was found to be dysregulated in prostate (89) and bladder (90) cancers, and associated with poor prognosis in both cancer types.

Our findings with differential expression of 5′ tRNA halves and 5′ YRNA fragments in OSCC patients is in alignment with previous studies with miRNAs (18). Volinia and colleagues (7) had identified 21 miRNAs commonly regulated in solid lung, breast, stomach, prostate, colon, and pancreatic tumors. In previous work of our group, we observed that four of these 21 miRNAs were also differentially regulated between healthy and tumor tissue in OSCC patients (18), suggesting that these miRNAs are important and consistently associated with different types of cancer. In the same way, a previous case-control study from our group identified several serum miRNAs deregulated in patients with HNSCC (17). Several of those were also deregulated in the tissue of OSCC (18), further suggesting these miRNAs as biomarkers for non-invasive diagnosis. Based on these, we suggest that besides miRNAs, the 5′ tRNA halves and 5′ YRNA fragments found deregulated in the current study should be further studied and can be targets not only in HNSCC, but other types of cancers as well.

In conclusion, we found that serum levels of 5′ tRNA halves and 5′ YRNA fragments changed in association with OSCC and that levels of 5′ tRNA halves changed in cancer tissues relatively to adjacent healthy tissues. These and comparable previous findings imply that 5′ tRNA halves and 5′ YRNA fragments may play a role in tumorigenesis. By additionally conducting weighted gene coexpression network analysis of 5′ tRNA halves, 5′ YRNA fragments, and miRNAs measured in the same samples and assessing the functional enrichment in a network of relevant overtargeted genes, we uncovered a potential novel mechanism by which coregulated modules of miRNAs and tRNA halves regulate OSCC. Our study has important limitations including the small sample size and the fact that all tumor samples covered a single TNM classification (i.e., pT2 N1 M0), which limits the generalizability of our findings and warrants validation in independent cohorts (ongoing efforts from our group and others). However, we were able to identify significant differences that underscore the important roles of novel sncRNAs such as 5′ tRNA-Val-CAC-2-1 half in OSSC and were able to experimentally validate our main claims from the functional network analysis. Of note, this is the first time, to our knowledge, that the specific molecular function of a 5′-tRNA half is specifically pinpointed in OSCC. This particular sncRNA is suggested to have potential for use as both, biomarker and therapeutic target in OSCC.

## Materials and Methods

### Patients

Tumor samples from five patients (2 males and 3 females, 64.4 ± 5.6 years old) diagnosed with HNSCC were used. All patients were qualified for the primary surgical resection by the multidisciplinary team from the institution. Recurrences and patients initially treated with other therapeutic approaches were excluded, as well as HPV positive tumors. All tumors were confirmed by a pathologist and have been collected intraoperatively as it was previously published (91, 92). All samples were classified based on the guidelines from the International Union Against Cancer using standard TNM classification. Three of the five samples were located at the floor of the mouth and the remaining two in the base of the tongue. All samples were classified as pT2 N1 M0. This study was carried out in accordance with the recommendations and approval by Institutional Review Board of the University of Medical Sciences in Poznan. All subjects gave written informed consent in accordance with the Declaration of Helsinki.

### Sample and Tissue Collection

Blood samples were collected prior surgical intervention and centrifuged for serum separation. Additionally, two separate tissue samples were collected during surgical resection, one from the tumor and one from a healthy mucosa adjacent to the tumor in the same patient as described previously (18). Samples were immediately frozen in liquid nitrogen and stored ay −80°C.

### RNA Isolation and Small RNA Library Construction

As previously described in reference (18), tissues samples were removed from the −80°C freezer and homogenized with Qiazol (Qiagen, Valencia, CA, USA) using 0.5 mm zirconium oxide beads in the Bullet Blender 24 (Next Advance, Averill Park, NY, USA). Total RNA was extracted using a commercial column purification system (miRNeasy Mini Kit, Qiagen) and on-column DNase treatment (RNase-free DNase Set, Qiagen) following manufacturer's instructions. Serum samples were extracted using the miRNEasy Serum/Plasma kit (Qiagen) following manufactures instructions but adjusting for an initial volume of 300 μL of serum. MicroRNA libraries were prepared using the TruSeq Small RNA Sample Preparation Kit (Illumina Inc., San Diego, CA, USA) following the manufacturer's instructions and adjusted by Matkovich et al. (93). Briefly, small RNAs from 1 μg of total RNA or from 300 μL of serum (not quantified) were ligated with 3′ and 5′ adapters, followed by reverse transcription to produce single stranded cDNAs. Samples were then amplified by PCR in 14 cycles (94°C for 30 s, 14 cycles of 94°C for 15 s, 62°C for 30 s, and 70°C for 15 s, and a final extension of 70°C for 5 min) using indexes to allow all individual libraries to be processed in a single flowcell lane during sequencing. The amplified libraries were size-selected and purified in a 6% agarose gel. The quantity and quality of miRNA libraries was determined using BioAnalyzer and RNA Nano Lab Chip Kit (Agilent Technologies, Santa Clara, CA, USA), and the samples were combined in a single microtube and submitted to sequencing on a HiSeq. 2500 instrument (Illumina Inc.) using the Illumina HiSeq v4 kit in a single read 50 bp (1 × 50) run.

### Quantitative Real Time PCR (qPCR)

Confirmation of tissue gene expression changes in genes targeted by cancer-associated miRNAs and tRNA halves was conducted by qPCR as described by Allen and collaborators (94) with some modifications. In summary, RNA extracted from tumor and normal adjacent tissue as described in previous section was converted to cDNA using the iScript cDNA synthesis kit (Bio-Rad) following the manufacturer instructions. qPCR reactions were set up in a MicroAmp^®^ Fast Optical 96-well reaction plate (Applied Biosystems) with 2 μL of diluted cDNA, 0.2 μL each of forward and reverse primer, 12.6 μL of nuclease free water, and 5 μL of Fast SYBR Green Master Mix (Applied Biosystems) per well. Amplification was performed in a 7900 HT Fast system (Applied Biosystems) at 95°C for 20 s, followed by 45 cycles of 1 s denaturation at 95°C and 20 s annealing/extension at 62°C. Primer sequences used were: FBXO31 (forward: 5′-GCCGTGAGGAGTATGGTGTTT-3′ and reverse: 5′-GTACATCCACCCGATGATGAAC-3′), WEE1 (forward: 5′-CTTGGGGACTTCTGCATGA-3′ and reverse: 5′-GCTTGGGGACTATCACCACT-3′), RB1 (forward: 5′-CTCTCGTCAGGCTTGAGTTTG-3′ and reverse: 5′-GACATCTCATCTAGGTCAACTGC-3′), and E2F1 (forward: 5′-CTACGTGACGTTCAGGACC-3′ and reverse: 5′-CCGGAGTTCCCGATCTAC-3′). β2-microglobulin (forward primer: 5′-GAGTATGCCTGCCGTGTGAA-3′ and reverse primer: 5′-CGGCATCTTCAAACCTCCAT-3′) was used as the housekeeping gene to normalize the qPCR data. Relative quantification was implemented using the 2^−ΔΔCt^ method for calculation of fold changes in gene expression. Statistical analysis using the Student's *t*-test was implemented on the log transformed fold change data.

### Bioinformatics and Statistical Analyses of the Circulating tRNA- and YRNA-Derived Small RNAs in Patients With and Without Cancer

The sequencing reads obtained from control and cancer serum samples were trimmed by removing adaptor sequences with FASTX-Toolkit (hannonlab.cshl.edu) and mapped to the GRCh38/hg38 human genome with Bowtie v1.1.2 [35]. The obtained aligned reads were analyzed to annotate and count the reads that map to tRNA genes from Genomic tRNA Database (95, 96), YRNA genes from the UCSC GENCODE v24 track (97), and non-coding RNA genes from Ensembl GRCh38 release 86.

To assess differences in the abundance of circulating tRNA- and YRNA-derived small RNAs between cancer and control groups, we analyzed the read count using the negative binomial model in the Bioconductor package edgeR (39). After normalization of the read counts with the trimmed mean of M-values (TMM) method, the differential expression was assessed by the exact test which is applicable to experiments with a single factor. *P*-values were adjusted for multiple testing using the Benjamini and Hochberg method to control the false discovery rate (FDR). Differences in expression were considered significant below an FDR of 5%.

### Bioinformatics and Statistical Analyses of tRNA- and YRNA-Derived Small RNAs in Tumor and Matched Normal Tissues

Sequencing reads from tissue of tumor-normal pairs were aligned to the GRCh38/hg38 human genome using the same parameters used for the analysis of the small RNA-Seq from the serum samples described above. To detect statistically significant differential expression of tRNA- and YRNA-derived small RNAs, read counts from tissue of tumor-normal pairs were analyzed with the Bioconductor package edgeR using a statistical test appropriate for paired designs (39). The test measures differential gene expression between tumor and normal tissue. To adjust for differences between the patients, the test uses an additive linear model with “Patient” as the blocking factor*. P*-values were adjusted for multiple testing using the Benjamini and Hochberg method to control the FDR. Differences in expression were considered significant below an FDR of 5%. Multi-dimensional scaling (MDS) analysis of the expression levels of 5′ tRNA-halves in serum and tissues was performed using the edgeR function plotMDS (39).

### Weighted Gene Coexpression Network Analysis (WGCNA)

Traditional genetic approaches focusing on the action of single genes have contributed to the identification of important candidate genes for many diseases, particularly monogenetic diseases. However, these approaches have limited effectiveness in identifying genes and other genetic features (e.g., sncRNAs) that contribute to complex diseases such as cancer. On the other hand, the analysis of entire gene and non-coding RNA networks using modern genomics and bioinformatic tools have proven invaluable for the identification of biological pathways underlying disease (98, 99). WGCNA is a network analysis tool based on a coexpression measure that describes the correlation among genetic features with similar patterns of expression. Those sets of features with similar (correlated) expression patterns are denominated modules and represent non-linear pathways of related biological functions. The general framework of WGCNA has been described in detail elsewhere (40, 41, 47).

In this study, WGCNA was implemented as previously reported by Nunez et al. (43) and Gorini et al. (42) with some modifications as described below. Normalized small RNA-Seq data for tissue (cancer vs. normal) 5′ tRNA halves, 5′ YRNA fragments (this work) and miRNA (18) were pooled into a single data table for WGCNA analysis (47) in the R biostatistical computing environment. WGCNA was performed on all tissue samples and included 540 genetic features (i.e., 5′ tRNA halves, 5′ YRNA fragments, and miRNAs) with median absolute deviation (MAD, a robust measure of variability) <0 (out of a total of 1,429 features). A soft power of β = 6 was chosen to produce a network with approximate scale-free topology (soft *R*^2^ = 0.8). Parameter deepSplit was set to 3. Because the number of genetic features in our analysis is lower than the number of genes usually detected in a microarray study (the data type for which WGCNA was originally developed), we reduced the minimum module size parameter to 10. All other parameters were used as defined by default. Gene modules corresponding to the branches cut off of the gene tree were labeled in unique colors. Unassigned genes were assigned to the Gray module. miRNA-overtargeted gene (validated interaction) networks were constructed for the Green module as described below. The trait-based gene significance (GS) measure is defined in WGCNA as the absolute value of the correlation between the expression profile of a specific node (genetic feature such as miRNA, tRNA half, or YRNA fragment in our case) and the sample trait (cancer vs. normal tissue). The higher the value of GS for a specific genetic feature, the more biologically significant the specific genetic feature is in the context of the specific trait. On the other hand, module membership, also known as eigengene-based connectivity, correlates the expression profile of the specific genetic feature with the module eigengene (equivalent to a representative expression pattern) of a given module. The higher the value of MM for a specific genetic feature, the higher the contribution of the specific genetic feature to the overall pattern and possibly function of the specific module (pathway). By focusing on modules with high correlation to sample traits of interest (cancer in our case), and subsequently identifying within those modules the genetic features with higher GS and MM, one can identify the more important pathways and genetic features contributing to the disease trait of interest.

### Functional Network Analysis: miRNA-Overtargeted Analysis, Network Construction, and Enrichment Analysis of Gene Ontology Annotations

Functional enrichment analysis was performed as previously described (48). Significantly overtargeted genes in a particular network are defined as those genes that experimentally demonstrate significantly higher number of interactions with select miRNAs than expected by chance (as determined by hypergeometric tests of network proportions compared to the respective proportions in the miRNA–target interaction universe/background) (48). By identifying enriched biological processes and functions for the significantly overtargeted genes, we could assign specific functions to the related module(s) and to novel genetic features with high module membership as calculated using WGCNA. This miRNA-overtargeting analysis approach is supported by our previous experiences and those from others indicating that global miRNA-driven regulatory events commonly occur in a coordinated/cooperative fashion (17, 20, 42, 43, 48–52). In short, significance of the overtargeting effect (significant higher number of miRNA-targeting events than expected by chance on a given gene) was assessed by comparing module-specific network proportions and relevant number of events against a collection of 100,000 simulated equivalent random networks. The list of validated targets supported by strong experimental evidence (i.e., reporter assay or Western blot) used for this analysis was downloaded from miRTarBase (file: miRTarBase_SE_WR.xls) using the SpidermiR package (100). Interaction networks were constructed using Cytoscape 3.5.1 (101). Enrichment of gene ontology annotations among sets of overtargeted genes was assessed using the GOCluster_Report function of the systemPipeR package (102) in the R environment.

## Data Availability Statement

Datasets in this study are available at: https://www.ncbi.nlm.nih.gov/bioproject with the accession BioProject: PRJNA561150.

## Ethics Statement

This study was carried out in accordance with the recommendations and approval by Institutional Review Board of the University of Medical Sciences in Poznan. All subjects gave written informed consent in accordance with the Declaration of Helsinki.

## Author Contributions

Individual contributions to the present work was as follows: JD, YN, and MM: conceptualization and methodology. JD, KB, TM, YN, AS, and BV: software. JD, YN, AS, BV, TS, KB, TM, HA, WS, PG, WG, and MM: validation, data curation, manuscript writing, reviewing, and editing. JD, YN, AS, and MM: formal analysis. JD, AS, BV, TS, KB, TM, HA, WS, PG, and WG: experimental investigation. JD, YN, PG, WG, and MM: resources. JD and MM: supervision, project administration, and funding acquisition.

### Conflict of Interest

The authors declare that the research was conducted in the absence of any commercial or financial relationships that could be construed as a potential conflict of interest.
