# Supplementary material for: Profiling of tRNA Halves and YRNA Fragments in Serum and Tissue From Oral Squamous Cell Carcinoma Patients Identify Key Role of 5′ tRNA-Val-CAC-2-1 Half
Source: Front Oncol. 2019 Sep 26;9:959. doi: 10.3389/fonc.2019.00959 (PMC6775249; doi:10.3389/fonc.2019.00959)
Supplement: Supplementary file 1 [file Data_Sheet_1.docx]

Supplementary Material

Profiling of tRNA halves and YRNA fragments in serum and tissue from oral squamous cell carcinoma patients identify key role of 5’ tRNA-Val-CAC-2-1 half

Joseph Dhahbi^1^*^†^, Yury O. Nunez Lopez^2^*^†^, Augusto Schneider^3^, Berta Victoria^4^, Tatiana Saccon^3,4^, Krish Bharat^1^, Thaddeus McClatchey^1^, Hani Atamna1^1^, Wojciech Scierski^5^, Pawel Golusinski^6,7,8^, Wojciech Golusinski^8^, Michal M. Masternak^4,7,8^*

^1^Department of Medical Education, California University of Science & Medicine, School of Medicine, San Bernardino, CA 92324, USA.

^2^Translational Research Institute for Metabolism and Diabetes, AdventHealth, Orlando, FL 32804, USA.

^3^Faculdade de Nutrição, Universidade Federal de Pelotas, Pelotas, RS, Brazil

^4^College of Medicine, Burnett School of Biomedical Sciences, University of Central Florida, Orlando, FL 32827, USA.

^5^Department of Otorhinolaryngology and Laryngological Oncology in Zabrze, Medical University of Silesia, Katowice, Poland.

^6^Department of Otolaryngology and Maxillofacial Surgery, University of Zielona Gora, Zielona Gora, Poland.

^7^Department of Biology and Environmental Studies, Poznan University of Medical Sciences, Poland.

^8^Department of Head and Neck Surgery, Poznan University of Medical Sciences, The Greater Poland Cancer Centre, Poznan, Poland.

^†^These authors contributed equally.

*** Correspondence:**Joseph Dhahbi ([DhahbiJ@calmedu.org](mailto:DhahbiJ@calmedu.org)), Yury O. Nunez Lopez ([Yury.Nunez-Lopez@adventhealth.com](mailto:Yury.Nunez-Lopez@adventhealth.com)), and Michal M. Masternak [Michal.Masternak@ucf.edu](mailto:Michal.Masternak@ucf.edu)

Keywords: 5’ tRNA halves; 5’ YRNA fragments; Oral Cancer; OSCC; small RNA-Seq; microRNA; Coexpression Network; WGCNA


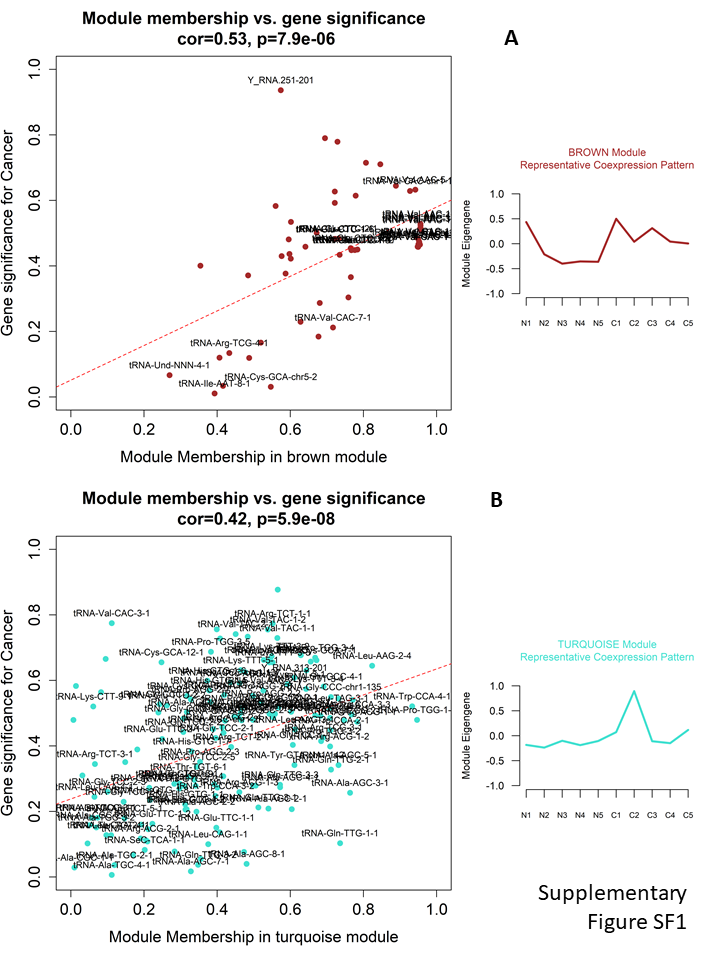


**Supplementary Figure SF1**. Correlation between module membership (MM) and gene significance (GS) and characteristic coexpression pattern (module eigengene) for additional modules of interest.


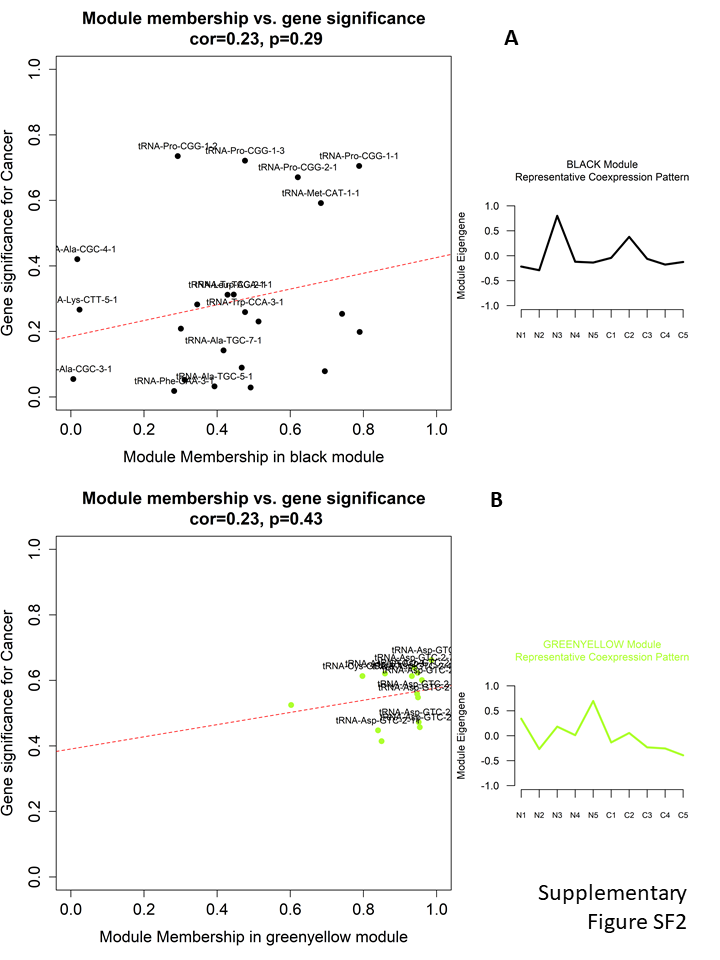


**Supplementary Figure SF2**. Correlation between module membership (MM) and gene significance (GS) and characteristic coexpression pattern (module eigengene) for additional modules of interest.

**Supplementary Table ST1**: Summary data for the coexpression green module.

| **Feature Index** | **Feature Name** | **Module Color** | **MM** | **GS** | **N tissue average CPM** | **C tissue average CPM** |
| --- | --- | --- | --- | --- | --- | --- |
| feature.1199 | tissue_hsa-miR-20a-5p | green | 0.96 | 0.82 | 87.07 | 214.73 |
| feature.1234 | tissue_hsa-miR-130b-3p | green | 0.95 | 0.79 | 49.40 | 90.77 |
| feature.1184 | tissue_hsa-miR-877-5p | green | 0.93 | 0.78 | 1.27 | 18.72 |
| feature.1227 | tissue_hsa-miR-24-2-5p | green | 0.91 | 0.68 | 25.24 | 51.30 |
| feature.1174 | tissue_hsa-miR-31-3p | green | 0.89 | 0.89 | 1.16 | 39.80 |
| feature.1220 | tissue_hsa-miR-17-5p | green | 0.89 | 0.69 | 114.70 | 226.81 |
| feature.1207 | tissue_hsa-miR-944 | green | 0.87 | 0.79 | 58.54 | 146.36 |
| feature.1194 | tissue_hsa-miR-92b-3p | green | 0.87 | 0.89 | 497.22 | 1465.13 |
| feature.1216 | tissue_hsa-miR-27a-5p | green | 0.87 | 0.79 | 25.85 | 56.42 |
| feature.1198 | tissue_hsa-miR-2467-5p | green | 0.85 | 0.72 | 1.52 | 10.68 |
| feature.202 | tissue_chr6_27280269_27280342_tRNA-Val-CAC-2-1:- | green | 0.83 | 0.67 | 26741.10 | 51928.05 |
| feature.1185 | tissue_hsa-miR-708-3p | green | 0.82 | 0.84 | 4.06 | 25.92 |
| feature.1271 | tissue_hsa-miR-708-5p | green | 0.81 | 0.68 | 72.19 | 111.85 |
| feature.1267 | tissue_hsa-miR-548av-5p | green | 0.81 | 0.62 | 8.63 | 16.22 |
| feature.1268 | tissue_hsa-miR-548k | green | 0.81 | 0.62 | 8.63 | 16.22 |
| feature.1252 | tissue_hsa-miR-452-5p | green | 0.80 | 0.60 | 24.39 | 53.63 |
| feature.1193 | tissue_hsa-miR-187-3p | green | 0.77 | 0.77 | 3.52 | 25.40 |
| feature.1233 | tissue_hsa-miR-1275 | green | 0.77 | 0.84 | 5.55 | 13.58 |
| feature.1259 | tissue_hsa-miR-182-5p | green | 0.76 | 0.51 | 7532.36 | 12360.82 |
| feature.1197 | tissue_hsa-miR-21-3p | green | 0.75 | 0.81 | 1366.33 | 3663.11 |
| feature.1176 | tissue_hsa-miR-424-5p | green | 0.75 | 0.88 | 2.14 | 39.75 |
| feature.1290 | tissue_hsa-miR-98-5p | green | 0.74 | 0.50 | 1064.56 | 1606.87 |
| feature.1254 | tissue_hsa-miR-130a-3p | green | 0.68 | 0.62 | 229.71 | 388.15 |
| feature.1172 | tissue_hsa-miR-31-5p | green | 0.68 | 0.87 | 128.27 | 2528.87 |
| feature.1335 | tissue_hsa-miR-200a-5p | green | 0.65 | 0.38 | 42.66 | 58.50 |
| feature.1350 | tissue_hsa-miR-183-5p | green | 0.64 | 0.23 | 2530.23 | 3392.75 |
| feature.1316 | tissue_hsa-miR-224-5p | green | 0.62 | 0.30 | 143.55 | 210.70 |
| feature.1369 | tissue_hsa-miR-16-5p | green | 0.59 | 0.28 | 4049.03 | 4663.38 |
| feature.1322 | tissue_hsa-miR-151a-3p | green | 0.58 | 0.57 | 2796.98 | 3643.48 |
| feature.1277 | tissue_hsa-let-7e-5p | green | 0.57 | 0.53 | 482.43 | 729.57 |
| feature.1294 | tissue_hsa-miR-455-3p | green | 0.52 | 0.55 | 32.97 | 49.87 |
| feature.91 | tissue_chr15_40593824_40593906_tRNA-Ser-GCT-4-2:- | green | 0.51 | 0.32 | 0.76 | 1.31 |
| feature.1188 | tissue_hsa-miR-18a-5p | green | 0.50 | 0.63 | 24.29 | 123.25 |
| feature.1266 | tissue_hsa-miR-421 | green | 0.50 | 0.39 | 37.45 | 64.03 |
| feature.1353 | tissue_hsa-miR-7974 | green | 0.49 | 0.34 | 11.95 | 16.26 |
| feature.1177 | tissue_hsa-miR-1910-5p | green | 0.46 | 0.83 | 0.00 | 13.45 |
| feature.1308 | tissue_hsa-miR-34c-5p | green | 0.45 | 0.21 | 44.49 | 73.04 |
| feature.206 | tissue_chr6_27334989_27335062_tRNA-Lys-TTT-6-1:- | green | 0.44 | 0.33 | 1.36 | 2.11 |
| feature.1275 | tissue_hsa-miR-99b-3p | green | 0.30 | 0.46 | 12.85 | 24.38 |
| feature.68 | tissue_chr11_122559946_122560019_tRNA-Lys-TTT-2-1:+ | green | 0.24 | 0.39 | 1.05 | 1.72 |
| feature.1402 | tissue_hsa-miR-99b-5p | green | 0.17 | 0.04 | 4909.19 | 5224.03 |
| feature.59 | tissue_chr10_5853710_5853783_tRNA-Val-TAC-3-1:- | green | 0.15 | 0.32 | 8.55 | 18.22 |
| feature.14 | tissue_chr1_143661181_143661253_tRNA-His-GTG-2-1:- | green | -0.13 | -0.14 | 1.05 | 1.35 |

**Supplementary Table ST2**: Summary data for the miRNA-overtargeting analysis.

| **Gene** | **Green Module Targeting miRNAs** | **Green Module Top miRNA Total** | **Targeting miRNAs in Universe** | **miRNA Total in Universe** | **hypergeometric P value** | **FDR** |
| --- | --- | --- | --- | --- | --- | --- |
| MDK | 2 | 10 | 2 | 234 | 0 | 0 |
| SIRPA | 2 | 10 | 2 | 234 | 0 | 0 |
| FBXO31 | 3 | 10 | 5 | 234 | 8.44E-06 | 0.00031 |
| TSG101 | 3 | 10 | 5 | 234 | 8.44E-06 | 0.00031 |
| ARL9 | 3 | 10 | 6 | 234 | 2.48E-05 | 0.00049 |
| EGR2 | 3 | 10 | 7 | 234 | 5.67E-05 | 0.00049 |
| ARHGEF7 | 2 | 10 | 3 | 234 | 5.69E-05 | 0.00049 |
| DAPK3 | 2 | 10 | 3 | 234 | 5.69E-05 | 0.00049 |
| ENPP5 | 2 | 10 | 3 | 234 | 5.69E-05 | 0.00049 |
| ETV1 | 2 | 10 | 3 | 234 | 5.69E-05 | 0.00049 |
| MAN1C1 | 2 | 10 | 3 | 234 | 5.69E-05 | 0.00049 |
| PAIP1 | 2 | 10 | 3 | 234 | 5.69E-05 | 0.00049 |
| PTPRO | 2 | 10 | 3 | 234 | 5.69E-05 | 0.00049 |
| RBL1 | 2 | 10 | 3 | 234 | 5.69E-05 | 0.00049 |
| SDHA | 2 | 10 | 3 | 234 | 5.69E-05 | 0.00049 |
| SLC25A28 | 2 | 10 | 3 | 234 | 5.69E-05 | 0.00049 |
| ZFYVE9 | 2 | 10 | 3 | 234 | 5.69E-05 | 0.00049 |
| PBXIP1 | 3 | 10 | 8 | 234 | 0.00011 | 0.00085 |
| POGZ | 3 | 10 | 8 | 234 | 0.00011 | 0.00085 |
| ABCA3 | 2 | 10 | 4 | 234 | 0.00022 | 0.00093 |
| BNIP2 | 2 | 10 | 4 | 234 | 0.00022 | 0.00093 |
| BTN3A1 | 2 | 10 | 4 | 234 | 0.00022 | 0.00093 |
| EPAS1 | 2 | 10 | 4 | 234 | 0.00022 | 0.00093 |
| HEXIM1 | 2 | 10 | 4 | 234 | 0.00022 | 0.00093 |
| HIST1H2AM | 2 | 10 | 4 | 234 | 0.00022 | 0.00093 |
| LIMK1 | 2 | 10 | 4 | 234 | 0.00022 | 0.00093 |
| MAP3K12 | 2 | 10 | 4 | 234 | 0.00022 | 0.00093 |
| PPP6R3 | 2 | 10 | 4 | 234 | 0.00022 | 0.00093 |
| PSD3 | 2 | 10 | 4 | 234 | 0.00022 | 0.00093 |
| STIL | 2 | 10 | 4 | 234 | 0.00022 | 0.00093 |
| STK11 | 2 | 10 | 4 | 234 | 0.00022 | 0.00093 |
| TBC1D15 | 2 | 10 | 4 | 234 | 0.00022 | 0.00093 |
| VLDLR | 2 | 10 | 4 | 234 | 0.00022 | 0.00093 |
| WDR82 | 2 | 10 | 4 | 234 | 0.00022 | 0.00093 |
| ZNFX1 | 2 | 10 | 4 | 234 | 0.00022 | 0.00093 |
| MBNL1 | 3 | 10 | 11 | 234 | 0.00049 | 0.00159 |
| MEF2D | 3 | 10 | 11 | 234 | 0.00049 | 0.00159 |
| ADAR | 2 | 10 | 5 | 234 | 0.00054 | 0.00159 |
| ARPC2 | 2 | 10 | 5 | 234 | 0.00054 | 0.00159 |
| CAMSAP1 | 2 | 10 | 5 | 234 | 0.00054 | 0.00159 |
| MORF4L2 | 2 | 10 | 5 | 234 | 0.00054 | 0.00159 |
| MUC17 | 2 | 10 | 5 | 234 | 0.00054 | 0.00159 |
| PKNOX1 | 2 | 10 | 5 | 234 | 0.00054 | 0.00159 |
| PLXNA1 | 2 | 10 | 5 | 234 | 0.00054 | 0.00159 |
| RBL2 | 2 | 10 | 5 | 234 | 0.00054 | 0.00159 |
| RBM10 | 2 | 10 | 5 | 234 | 0.00054 | 0.00159 |
| RNF145 | 2 | 10 | 5 | 234 | 0.00054 | 0.00159 |
| RPA2 | 2 | 10 | 5 | 234 | 0.00054 | 0.00159 |
| STRBP | 2 | 10 | 5 | 234 | 0.00054 | 0.00159 |
| TXN2 | 2 | 10 | 5 | 234 | 0.00054 | 0.00159 |
| TP53INP1 | 3 | 10 | 12 | 234 | 0.00072 | 0.00206 |
| ATP5G3 | 2 | 10 | 6 | 234 | 0.00106 | 0.00235 |
| CRIM1 | 2 | 10 | 6 | 234 | 0.00106 | 0.00235 |
| CTSA | 2 | 10 | 6 | 234 | 0.00106 | 0.00235 |
| FOXJ3 | 2 | 10 | 6 | 234 | 0.00106 | 0.00235 |
| GRB10 | 2 | 10 | 6 | 234 | 0.00106 | 0.00235 |
| IL8 | 2 | 10 | 6 | 234 | 0.00106 | 0.00235 |
| ITGB8 | 2 | 10 | 6 | 234 | 0.00106 | 0.00235 |
| KANSL1 | 2 | 10 | 6 | 234 | 0.00106 | 0.00235 |
| MLXIP | 2 | 10 | 6 | 234 | 0.00106 | 0.00235 |
| PAPOLA | 2 | 10 | 6 | 234 | 0.00106 | 0.00235 |
| POGK | 2 | 10 | 6 | 234 | 0.00106 | 0.00235 |
| PPARG | 2 | 10 | 6 | 234 | 0.00106 | 0.00235 |
| SURF4 | 2 | 10 | 6 | 234 | 0.00106 | 0.00235 |
| UBE2C | 2 | 10 | 6 | 234 | 0.00106 | 0.00235 |
| WAC | 2 | 10 | 6 | 234 | 0.00106 | 0.00235 |
| ADARB1 | 2 | 10 | 7 | 234 | 0.00182 | 0.00363 |
| EGLN3 | 2 | 10 | 7 | 234 | 0.00182 | 0.00363 |
| PPP2R1A | 2 | 10 | 7 | 234 | 0.00182 | 0.00363 |
| RBM12B | 2 | 10 | 7 | 234 | 0.00182 | 0.00363 |
| RNF41 | 2 | 10 | 7 | 234 | 0.00182 | 0.00363 |
| Sept2 | 2 | 10 | 7 | 234 | 0.00182 | 0.00363 |
| SSRP1 | 2 | 10 | 7 | 234 | 0.00182 | 0.00363 |
| KMT2D | 3 | 10 | 16 | 234 | 0.00243 | 0.00473 |
| RB1 | 3 | 10 | 16 | 234 | 0.00243 | 0.00473 |
| CCDC47 | 2 | 10 | 8 | 234 | 0.00284 | 0.00482 |
| JAK1 | 2 | 10 | 8 | 234 | 0.00284 | 0.00482 |
| MTMR3 | 2 | 10 | 8 | 234 | 0.00284 | 0.00482 |
| NAP1L1 | 2 | 10 | 8 | 234 | 0.00284 | 0.00482 |
| NFAT5 | 2 | 10 | 8 | 234 | 0.00284 | 0.00482 |
| PTBP1 | 2 | 10 | 8 | 234 | 0.00284 | 0.00482 |
| RPS27 | 2 | 10 | 8 | 234 | 0.00284 | 0.00482 |
| SLC25A3 | 2 | 10 | 8 | 234 | 0.00284 | 0.00482 |
| SLC25A5 | 2 | 10 | 8 | 234 | 0.00284 | 0.00482 |
| SMG1 | 2 | 10 | 8 | 234 | 0.00284 | 0.00482 |
| ZNF598 | 2 | 10 | 8 | 234 | 0.00284 | 0.00482 |
| LDLR | 3 | 10 | 17 | 234 | 0.00311 | 0.00515 |
| TGFBR2 | 3 | 10 | 17 | 234 | 0.00311 | 0.00515 |
| NACC2 | 2 | 10 | 9 | 234 | 0.00416 | 0.00653 |
| NONO | 2 | 10 | 9 | 234 | 0.00416 | 0.00653 |
| PPARA | 2 | 10 | 9 | 234 | 0.00416 | 0.00653 |
| RAB23 | 2 | 10 | 9 | 234 | 0.00416 | 0.00653 |
| RPL21 | 2 | 10 | 9 | 234 | 0.00416 | 0.00653 |
| ANKRD52 | 2 | 10 | 10 | 234 | 0.00581 | 0.00832 |
| CDK19 | 2 | 10 | 10 | 234 | 0.00581 | 0.00832 |
| DDX5 | 2 | 10 | 10 | 234 | 0.00581 | 0.00832 |
| FBXO28 | 2 | 10 | 10 | 234 | 0.00581 | 0.00832 |
| MAPK9 | 2 | 10 | 10 | 234 | 0.00581 | 0.00832 |
| PPP2R2A | 2 | 10 | 10 | 234 | 0.00581 | 0.00832 |
| PTP4A1 | 2 | 10 | 10 | 234 | 0.00581 | 0.00832 |
| RAB1B | 2 | 10 | 10 | 234 | 0.00581 | 0.00832 |
| RUNX3 | 2 | 10 | 10 | 234 | 0.00581 | 0.00832 |
| E2F1 | 3 | 10 | 21 | 234 | 0.00716 | 0.01014 |
| ARHGAP12 | 2 | 10 | 11 | 234 | 0.00780 | 0.01027 |
| ILF3 | 2 | 10 | 11 | 234 | 0.00780 | 0.01027 |
| NCOA3 | 2 | 10 | 11 | 234 | 0.00780 | 0.01027 |
| NUCKS1 | 2 | 10 | 11 | 234 | 0.00780 | 0.01027 |
| PGAM1 | 2 | 10 | 11 | 234 | 0.00780 | 0.01027 |
| RAB5B | 2 | 10 | 11 | 234 | 0.00780 | 0.01027 |
| RUNX1 | 2 | 10 | 11 | 234 | 0.00780 | 0.01027 |
| TCEAL1 | 2 | 10 | 11 | 234 | 0.00780 | 0.01027 |
| WEE1 | 3 | 10 | 22 | 234 | 0.00855 | 0.01115 |
| STAT3 | 3 | 10 | 23 | 234 | 0.01013 | 0.01309 |
| GATA6 | 2 | 10 | 13 | 234 | 0.01291 | 0.01597 |
| PHLPP1 | 2 | 10 | 13 | 234 | 0.01291 | 0.01597 |
| RAN | 2 | 10 | 13 | 234 | 0.01291 | 0.01597 |
| SON | 2 | 10 | 13 | 234 | 0.01291 | 0.01597 |
| ZBTB4 | 2 | 10 | 13 | 234 | 0.01291 | 0.01597 |
| LARP1 | 2 | 10 | 14 | 234 | 0.01605 | 0.01953 |
| SMAD3 | 2 | 10 | 14 | 234 | 0.01605 | 0.01953 |
| APP | 2 | 10 | 15 | 234 | 0.01960 | 0.02326 |
| BMPR2 | 2 | 10 | 15 | 234 | 0.01960 | 0.02326 |
| THBS1 | 2 | 10 | 15 | 234 | 0.01960 | 0.02326 |
| CCND2 | 3 | 10 | 28 | 234 | 0.02094 | 0.02466 |
| EIF4G2 | 2 | 10 | 16 | 234 | 0.02356 | 0.02730 |
| MMP2 | 2 | 10 | 16 | 234 | 0.02356 | 0.02730 |
| AGO1 | 4 | 10 | 46 | 234 | 0.02758 | 0.03163 |
| HNRNPUL1 | 2 | 10 | 17 | 234 | 0.02794 | 0.03163 |
| SMAD7 | 2 | 10 | 17 | 234 | 0.02794 | 0.03163 |
| SMAD4 | 3 | 10 | 31 | 234 | 0.03008 | 0.03378 |
| HIF1A | 2 | 10 | 18 | 234 | 0.03275 | 0.03595 |
| HIST2H4B | 2 | 10 | 18 | 234 | 0.03275 | 0.03595 |
| TUBB | 2 | 10 | 18 | 234 | 0.03275 | 0.03595 |
| FLNA | 2 | 10 | 19 | 234 | 0.03799 | 0.04139 |
| HUWE1 | 2 | 10 | 20 | 234 | 0.04364 | 0.04720 |
| PTEN | 4 | 10 | 53 | 234 | 0.04974 | 0.05301 |
| FASN | 2 | 10 | 21 | 234 | 0.04972 | 0.05301 |
| BCL2L11 | 2 | 10 | 24 | 234 | 0.07046 | 0.07455 |
| E2F3 | 2 | 10 | 26 | 234 | 0.08629 | 0.09064 |
| CDKN1A | 2 | 10 | 27 | 234 | 0.09479 | 0.09815 |
| DNMT1 | 2 | 10 | 27 | 234 | 0.09479 | 0.09815 |
| BCL2 | 3 | 10 | 45 | 234 | 0.10310 | 0.10600 |
| MYC | 2 | 10 | 29 | 234 | 0.11286 | 0.11523 |
| EEF1A1 | 2 | 10 | 34 | 234 | 0.16385 | 0.16613 |
| CCND1 | 2 | 10 | 36 | 234 | 0.18627 | 0.18755 |
| VEGFA | 2 | 10 | 37 | 234 | 0.19785 | 0.19785 |

**Supplementary Table ST3**: KEGG pathway enrichment analysis for the top 10 miRNA-overtargeted gene network.

| **KEGG ID** | **Pathway Name** | **Genes Found** | **Genes Pathway** | **%** | **P value** | **Adjusted P value** | **Gene IDs** |
| --- | --- | --- | --- | --- | --- | --- | --- |
| 4350 | TGF-beta signaling pathway | 9 | 80 | 11.3 | 2.98E-07 | 3.33E-05 | RBL1, ZFYVE9, PPP2R1A, TGFBR2, SMAD3, BMPR2, THBS1, SMAD7, SMAD4 |
| 5212 | Pancreatic cancer | 8 | 71 | 11.3 | 1.10E-06 | 9.20E-05 | RB1, JAK1, TGFBR2, MAPK9, E2F1, STAT3, SMAD3, SMAD4 |
| 4659 | Th17 cell differentiation | 8 | 73 | 11.0 | 1.39E-06 | 9.32E-05 | JAK1, TGFBR2, MAPK9, RUNX1, STAT3, SMAD3, SMAD4, HIF1A |
| 5166 | Human T-cell leukemia virus 1 infection | 11 | 171 | 6.4 | 8.07E-06 | 4.51E-04 | EGR2, RB1, JAK1, SLC25A5, TGFBR2, MAPK9, E2F1, RAN, SMAD3, CCND2, SMAD4 |
| 5200 | Pathways in cancer | 17 | 420 | 4.0 | 2.10E-05 | 1.01E-03 | DAPK3, EPAS1, PPARG, EGLN3, RB1, JAK1, TGFBR2, MAPK9, E2F1, NCOA3, RUNX1, STAT3, SMAD3, CCND2, MMP2, SMAD4, HIF1A |
| 5219 | Bladder cancer | 5 | 39 | 12.8 | 2.55E-05 | 1.07E-03 | DAPK3, RB1, E2F1, THBS1, MMP2 |
| 4068 | FoxO signaling pathway | 8 | 111 | 7.2 | 4.25E-05 | 1.30E-03 | STK11, RBL2, TGFBR2, MAPK9, STAT3, SMAD3, CCND2, SMAD4 |
| 4110 | Cell cycle | 8 | 111 | 7.2 | 4.25E-05 | 1.30E-03 | RBL1, RBL2, RB1, E2F1, WEE1, SMAD3, CCND2, SMAD4 |
| 5161 | Hepatitis B | 9 | 136 | 6.6 | 3.49E-05 | 1.30E-03 | EGR2, RB1, JAK1, TGFBR2, MAPK9, E2F1, STAT3, SMAD3, SMAD4 |
| 5160 | Hepatitis C | 8 | 121 | 6.6 | 8.28E-05 | 2.31E-03 | PPP2R1A, RB1, JAK1, LDLR, PPARA, PPP2R2A, E2F1, STAT3 |
| 5220 | Chronic myeloid leukemia | 6 | 75 | 8.0 | 1.38E-04 | 3.55E-03 | RB1, TGFBR2, E2F1, RUNX1, SMAD3, SMAD4 |
| 4390 | Hippo signaling pathway | 8 | 134 | 6.0 | 1.79E-04 | 4.27E-03 | PPP2R1A, TGFBR2, PPP2R2A, SMAD3, BMPR2, CCND2, SMAD7, SMAD4 |
| 4218 | Cellular senescence | 8 | 139 | 5.8 | 2.34E-04 | 5.23E-03 | RBL1, RBL2, RB1, SLC25A5, TGFBR2, E2F1, SMAD3, CCND2 |
| 4144 | Endocytosis | 9 | 178 | 5.1 | 3.12E-04 | 6.53E-03 | TSG101, ZFYVE9, PSD3, ARPC2, RNF41, LDLR, TGFBR2, RAB5B, SMAD3 |
| 4933 | AGE-RAGE signaling pathway in diabetic complications | 6 | 93 | 6.5 | 5.15E-04 | 0.0101 | TGFBR2, MAPK9, STAT3, SMAD3, MMP2, SMAD4 |
| 3015 | mRNA surveillance pathway | 5 | 75 | 6.7 | 9.72E-04 | 0.0181 | WDR82, PAPOLA, PPP2R1A, SMG1, PPP2R2A |
| 5203 | Viral carcinogenesis | 8 | 176 | 4.5 | 1.25E-03 | 0.0213 | EGR2, RBL1, RBL2, RB1, JAK1, STAT3, CCND2, HIST2H4B |
| 5165 | Human papillomavirus infection | 10 | 257 | 3.9 | 1.36E-03 | 0.0217 | RBL1, RBL2, ITGB8, PPP2R1A, RB1, JAK1, PPP2R2A, E2F1, THBS1, CCND2 |
| 1522 | Endocrine resistance | 5 | 86 | 5.8 | 1.96E-03 | 0.0282 | RB1, MAPK9, E2F1, NCOA3, MMP2 |
| 5169 | Epstein-Barr virus infection | 7 | 152 | 4.6 | 2.02E-03 | 0.0282 | RB1, JAK1, MAPK9, RUNX3, E2F1, STAT3, CCND2 |

**Supplementary Table ST4**: Gene ontology annotation enrichment analysis for the top 10 miRNA-overtargeted gene network.

| **GO ID** | **Node Size** | **Sample Match** | **P hyper** | **P adj** | **Term** | **Genes ID** |
| --- | --- | --- | --- | --- | --- | --- |
| GO:0030154 | 4154 | 16 | 9.46E-09 | 5.96E-07 | cell differentiation | MEF2D WEE1 LDLR AGO1 PBXIP1 E2F1 TSG101 KMT2D TP53INP1 MBNL1 RB1 STAT3 SMAD4 FBXO31 EGR2 TGFBR2 |
| GO:0048869 | 4347 | 16 | 1.91E-08 | 1.20E-06 | cellular developmental process | MEF2D WEE1 LDLR AGO1 PBXIP1 E2F1 TSG101 KMT2D TP53INP1 MBNL1 RB1 STAT3 SMAD4 FBXO31 EGR2 TGFBR2 |
| GO:2000045 | 166 | 5 | 5.66E-07 | 3.56E-05 | regulation of G1/S transition of mitotic cell cycle | WEE1 E2F1 CCND2 RB1 FBXO31 |
| GO:0031328 | 1979 | 11 | 5.72E-07 | 3.61E-05 | positive regulation of cellular biosynthetic process | MEF2D LDLR AGO1 E2F1 TSG101 KMT2D TP53INP1 RB1 STAT3 SMAD4 EGR2 |
| GO:0009891 | 2013 | 11 | 6.81E-07 | 4.29E-05 | positive regulation of biosynthetic process | MEF2D LDLR AGO1 E2F1 TSG101 KMT2D TP53INP1 RB1 STAT3 SMAD4 EGR2 |
| GO:0010468 | 4615 | 15 | 7.32E-07 | 4.61E-05 | regulation of gene expression | MEF2D LDLR AGO1 PBXIP1 E2F1 TSG101 POGZ KMT2D TP53INP1 MBNL1 RB1 STAT3 SMAD4 EGR2 TGFBR2 |
| GO:0051173 | 3175 | 13 | 7.92E-07 | 4.99E-05 | positive regulation of nitrogen compound metabolic process | MEF2D LDLR AGO1 E2F1 TSG101 KMT2D TP53INP1 CCND2 RB1 STAT3 SMAD4 EGR2 TGFBR2 |
| GO:1903508 | 1600 | 10 | 8.90E-07 | 5.61E-05 | positive regulation of nucleic acid-templated transcription | MEF2D AGO1 E2F1 TSG101 KMT2D TP53INP1 RB1 STAT3 SMAD4 EGR2 |
| GO:1902680 | 1601 | 10 | 8.95E-07 | 5.64E-05 | positive regulation of RNA biosynthetic process | MEF2D AGO1 E2F1 TSG101 KMT2D TP53INP1 RB1 STAT3 SMAD4 EGR2 |
| GO:1902806 | 185 | 5 | 9.67E-07 | 6.09E-05 | regulation of cell cycle G1/S phase transition | WEE1 E2F1 CCND2 RB1 FBXO31 |
| GO:0031325 | 3300 | 13 | 1.26E-06 | 7.93E-05 | positive regulation of cellular metabolic process | MEF2D LDLR AGO1 E2F1 TSG101 KMT2D TP53INP1 CCND2 RB1 STAT3 SMAD4 EGR2 TGFBR2 |
| GO:0010604 | 3301 | 13 | 1.26E-06 | 7.96E-05 | positive regulation of macromolecule metabolic process | MEF2D LDLR AGO1 E2F1 TSG101 KMT2D TP53INP1 CCND2 RB1 STAT3 SMAD4 EGR2 TGFBR2 |
| GO:0051254 | 1684 | 10 | 1.43E-06 | 9.02E-05 | positive regulation of RNA metabolic process | MEF2D AGO1 E2F1 TSG101 KMT2D TP53INP1 RB1 STAT3 SMAD4 EGR2 |
| GO:0042127 | 1702 | 10 | 1.58E-06 | 9.95E-05 | regulation of cell proliferation | MEF2D E2F1 TSG101 KMT2D TP53INP1 CCND2 RB1 STAT3 SMAD4 TGFBR2 |
| GO:0045595 | 1816 | 10 | 2.87E-06 | 0.000181 | regulation of cell differentiation | LDLR AGO1 E2F1 KMT2D TP53INP1 RB1 STAT3 SMAD4 FBXO31 TGFBR2 |
| GO:0009893 | 3547 | 13 | 2.98E-06 | 0.000187 | positive regulation of metabolic process | MEF2D LDLR AGO1 E2F1 TSG101 KMT2D TP53INP1 CCND2 RB1 STAT3 SMAD4 EGR2 TGFBR2 |
| GO:0051171 | 6055 | 16 | 2.99E-06 | 0.000188 | regulation of nitrogen compound metabolic process | MEF2D LDLR AGO1 PBXIP1 E2F1 TSG101 POGZ KMT2D TP53INP1 CCND2 MBNL1 RB1 STAT3 SMAD4 EGR2 TGFBR2 |
| GO:0071930 | 3 | 2 | 3.01E-06 | 0.000189 | negative regulation of transcription involved in G1/S transition of mitotic cell cycle | E2F1 RB1 |
| GO:1904761 | 3 | 2 | 3.01E-06 | 0.000189 | negative regulation of myofibroblast differentiation | TP53INP1 RB1 |
| GO:0007399 | 2334 | 11 | 3.05E-06 | 0.000192 | nervous system development | MEF2D WEE1 LDLR E2F1 MBNL1 RB1 STAT3 SMAD4 FBXO31 EGR2 TGFBR2 |
| GO:0010557 | 1866 | 10 | 3.69E-06 | 0.000232 | positive regulation of macromolecule biosynthetic process | MEF2D AGO1 E2F1 TSG101 KMT2D TP53INP1 RB1 STAT3 SMAD4 EGR2 |
| GO:0044260 | 8733 | 18 | 3.78E-06 | 0.000238 | cellular macromolecule metabolic process | MEF2D WEE1 LDLR AGO1 PBXIP1 E2F1 TSG101 POGZ KMT2D TP53INP1 CCND2 MBNL1 RB1 STAT3 SMAD4 FBXO31 EGR2 TGFBR2 |
| GO:0051093 | 1037 | 8 | 3.85E-06 | 0.000243 | negative regulation of developmental process | LDLR AGO1 E2F1 TP53INP1 RB1 STAT3 SMAD4 TGFBR2 |
| GO:0045935 | 1905 | 10 | 4.46E-06 | 0.000281 | positive regulation of nucleobase-containing compound metabolic process | MEF2D AGO1 E2F1 TSG101 KMT2D TP53INP1 RB1 STAT3 SMAD4 EGR2 |
| GO:0080090 | 6228 | 16 | 4.56E-06 | 0.000288 | regulation of primary metabolic process | MEF2D LDLR AGO1 PBXIP1 E2F1 TSG101 POGZ KMT2D TP53INP1 CCND2 MBNL1 RB1 STAT3 SMAD4 EGR2 TGFBR2 |
| GO:0060255 | 6265 | 16 | 4.99E-06 | 0.000314 | regulation of macromolecule metabolic process | MEF2D LDLR AGO1 PBXIP1 E2F1 TSG101 POGZ KMT2D TP53INP1 CCND2 MBNL1 RB1 STAT3 SMAD4 EGR2 TGFBR2 |
| GO:0031323 | 6283 | 16 | 5.21E-06 | 0.000328 | regulation of cellular metabolic process | MEF2D LDLR AGO1 PBXIP1 E2F1 TSG101 POGZ KMT2D TP53INP1 CCND2 MBNL1 RB1 STAT3 SMAD4 EGR2 TGFBR2 |
| GO:2000134 | 116 | 4 | 5.26E-06 | 0.000331 | negative regulation of G1/S transition of mitotic cell cycle | WEE1 E2F1 RB1 FBXO31 |
| GO:0000082 | 262 | 5 | 5.34E-06 | 0.000336 | G1/S transition of mitotic cell cycle | WEE1 E2F1 CCND2 RB1 FBXO31 |
| GO:0032502 | 6296 | 16 | 5.37E-06 | 0.000338 | developmental process | MEF2D WEE1 LDLR AGO1 PBXIP1 E2F1 TSG101 KMT2D TP53INP1 MBNL1 RB1 STAT3 SMAD4 FBXO31 EGR2 TGFBR2 |
